# The ubiquitin E3 ligase TRIM27 emerges as a new player in mitophagy

**DOI:** 10.1080/27694127.2022.2164089

**Published:** 2023-01-05

**Authors:** Anne Kristin McLaren Berge, Juncal Garcia-Garcia, Eva Sjøttem, Hallvard Lauritz Olsvik

**Affiliations:** a Department of Medical Biology, Autophagy Research Group, University of Tromsø–The Arctic University of Norway, Norway; b Department of Molecular Cell Biology, Institute for Cancer Research, Oslo University Hospital, Norway

**Keywords:** E3 ligase, mitophagy, SQSTM1/p62, TBK1, TRIM

## Abstract

Mitochondria are the center for energy production, cell fate determination and synthesis of essential biomolecules in cells. Hence, mitochondrial quality control mechanisms are essential for cellular health. Failure of these control mechanisms may lead to damaged mitochondria that represent a threat to cell survival. Mitophagy is a selective autophagy process that removes damaged mitochondria through lysosomal degradation. The triggering of mitophagy can be either ubiquitin dependent or ubiquitin independent. Ubiquitin-dependent mitophagy relies on ubiquitin as a signal on the surface of dysfunctional mitochondria. PRKN/PARKIN is the ubiquitin E3 ligase of the well described PINK1-PRKN-dependent mitophagy. However, other ubiquitin-dependent mitophagy pathways that are independent of PRKN are emerging, but little is known about which ubiquitin E3 ligases are implicated. We shall here discuss our recent identification of the ubiquitin E3 ligase TRIM27 (tripartite motif containing 27) as a player in PINK1-PRKN-independent mitophagy. We will focus on the concerted action of TRIM27, the autophagy receptor SQSTM1/p62 and TBK1 (TANK binding kinase 1), which leads to mitochondrial clustering and enhanced mitophagy. We propose a model where a TRIM27-SQSTM1/p62-TBK1 pathway acts as an alternative or compensatory pathway for the PINK1-PRKN pathway to induce ubiquitin-dependent mitophagy.

## Main part

The tripartite family of proteins (TRIMs) constitute a large family of E3 ligases that mediate ubiquitination of a plethora of substrates. They are important players in several cellular processes, including innate immunity, macroautophagy/autophagy, and carcinogenesis. TRIM27 was originally identified as an RFPL/Rfp-RET fusion protein playing a vital role in RFPL-RET-induced oncogenesis. Later studies implicated TRIM27 ubiquitination activity in various signaling pathways, regulating cell proliferation, differentiation and apoptosis. TRIM27 is abnormally expressed in many types of cancer, and high TRIM27 expression is associated with worse clinicopathological features and a poor prognosis.

In an earlier screen performed to identify TRIM proteins that were localized in the lysosome, we found TRIM27 to be a putative player in autophagy. In our recent study, we confirmed that TRIM27 is an autophagic substrate [1], and we found that its degradation relies on the canonical autophagy pathway and the sequestosome-like autophagy receptors. TRIM27 does not act as autophagy receptor itself, as it is not degraded in HeLa penta knockout cells that do not express SQSTM1/p62, NBR1, CALCOCO2/NDP52, OPTN and TAX1BP1. Re-introduction of SQSTM1/p62 into the HeLa penta knockout cells is sufficient to restore autophagic degradation of TRIM27, confirming that TRIM27 is a cargo for the sequestosome-like autophagy receptors. Moreover, we identified TRIM27 to be a direct interaction partner of SQSTM1/p62. To test if TRIM27 is implicated in autophagy processes, we established *TRIM27* knockout cells and reconstituted these cells with inducible expression of EGFP-TRIM27. Surprisingly, imaging of the cells expressing reconstituted EGFP-TRIM27 revealed that the positioning and morphology of mitochondria is affected. Perinuclear mitochondrial clustering is detected both by quantitative immunohistochemical assays, and by qualitative correlative electron microscopy assays. SQSTM1/p62 is well recognized as a mediator of mitochondrial clustering. Based on our observations of direct interaction and colocalization of TRIM27 and SQSTM1/p62, we speculated whether SQSTM1/p62 is implicated in the TRIM27-mediated mitochondrial clustering. In cells overexpressing TRIM27 we observed SQSTM1/p62 to be enriched on the clustered mitochondria, and we do find TRIM27-mediated mitochondria clustering to be alleviated in *SQSTM1/p62* knockout cells.

Other studies have shown that efficient recruitment of SQSTM1/p62 to dysfunctional mitochondria is dependent on active TBK1. TBK1 is transiently recruited to polyubiquitinated mitochondria where it is activated by autophosphorylation. TBK1 promotes binding of SQSTM1/p62 to ubiquitinated substrates by phosphorylation events. Importantly, TBK1 interacts directly with TRIM27 via their coiled-coil domains. This prompted us to investigate if TBK1 acts in concert with TRIM27 and SQSTM1/p62 in the induction of mitochondrial clustering. Our data show strong colocalization of TRIM27 and phosphorylated TBK1 in cytoplasmic puncta, in addition to enrichment of phosphorylated TBK1 within the mitochondria clusters. Inhibition of TBK1 with the inhibitor MRT67307 in EGFP-TRIM27-expressing cells leads to less clustering of mitochondria. This was supported by cellular fractionation analyses, showing a larger amount of phosphorylated TBK1 in the mitochondria fraction isolated from TRIM27-expressing cells compared to *TRIM27* knockout cells

These observations suggested that TRIM27 can be involved in mitophagy, as clustering of mitochondria is involved in NLRP3 agonist-induced mitophagy and mitophagy in leukemia cells with the aid of the autophagy receptor SQSTM1/p62. Other studies have shown that depolarization-dependent mitophagy is dependent on TBK1. Activation of TBK1 promotes phosphorylation and efficient recruitment of SQSTM1/p62, facilitating mitophagy. Because mitophagy is a rare happening in cells grown under normal conditions, we induced mitophagy by co-overexpression of the mitophagy receptor FKBP8 and LC3A. The level of mitophagy was measured using double-tagged mCherry-EGFP-OMP25TM. We found that mitophagy is reduced in *TRIM27* knockout cells compared to WT cells, whereas re-introduction of TRIM27 in the knockout cells highly facilitates mitophagy. Moreover, inhibition of TBK1 with MRT67307 strongly reduces mitophagy both in WT cells and in those reconstituted with TRIM27. In *TRIM27* knockout cells however, TBK1 inhibition has no effect on mitophagy, thus showing TRIM27 to be necessary for TBK1-mediated mitophagy induced by FKBP8-LC3A.

**Figure 1.**
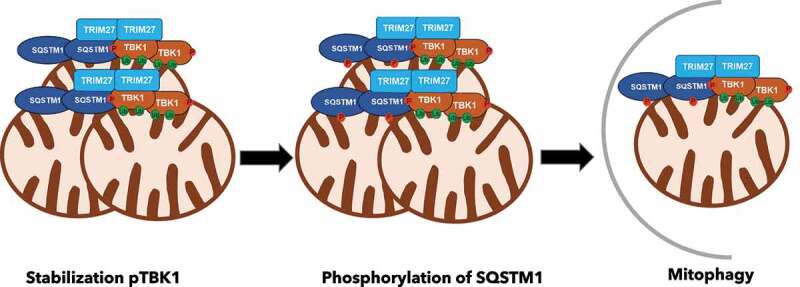
TRIM27-TBK1-SQSTM1/p62 complex assembly and induction of mitophagy. TRIM27 close to mitochondria recruit TBK1, leading to its activation and stabilization. Active TBK1 phosphorylates SQSTM1/p62, enhancing the affinity of SQSTM1/p62 for ubiquitinated substrates on the mitochondrial outer membrane leading to mitochondrial clustering and mitophagy.

To summarize, our data support a model in which TRIM27 via direct interactions and ubiquitination events, recruits SQSTM1/p62 and TBK1 to cytoplasmic bodies that are closely associated with mitochondria. Local enrichment of TBK1 leads to its activation by autophosphorylation. TBK1 phosphorylates SQSTM1/p62 increasing its affinity for ubiquitinated substrates. This TRIM27-SQSTM1/p62-TBK1 axis facilitates mitochondrial clustering and mitophagy (Fig. 1). TRIM27 expression level is elevated in several types of cancer. Dysfunction of mitophagy is closely connected to tumorigenesis and tumor development. In cancer cells, mitophagy improves the survival of tumor cells and supports the metastasis process. An intriguing question to address then, is if the oncogenic potential of TRIM27 can at least partially be due to its role as a promoter of mitophagy, supporting survival and metastasis of cancer cells.
